# “American Hookworm”—Uncinariasis

**Published:** 1908-06

**Authors:** A. G. Fort

**Affiliations:** Lumpkin, Ga.


### 'AMERICAN HOOKWORM"—UNCINARIASIS.*

### DEDUCTIONS DRAWN FROM TREATMENT OF 408 CASES.

BY A. G. FORT, PH. B., M. D., LUMPKIN, GA.

Uncinariasis is a specific disease caused by the presence of the Necator Americanus in the intestine and characterized by a progressive anemia, various nervous disturbances, weakness and intestinal disorders; common to temperate, tropical and sub-tropical climates and usually easily cured by removal of the parasites.

We have but to refer to the transactions of the Medical Association of Georgia for 1903 and 1904 to learn how recent is the discovery of this intesitnal parasite in our State. Since the articles of Dr. H. F. Harris in 1903 and Dr. Claude A. Smith in 1904, thousands have been treated in Georgia for this malady. While this is true, yet the relative number of physicians who recognize and treat it is indeed small.

Instead of thousands being treated, tens of thousands should have received treatment.

Doubtless this condition has existed in Georgia for numbers of years, yet it has been recognized as a disease only for the last six years.

Reports have come from all South Georgia of its presence. In Stewart county, southwest Georgia, I have, since April, 1904, treated 408 cases. If this is true of a hilly section of the southwestern part of the State it must be true of the moist, flat, warm sections.

All references in this article to the Hookworm apply to the one variety "Necator Americanus," described by Dr Stiles, who gave it this name, as follows:

"Uncinaria—Body Cylindrical, somewhat attenuated anteriorily. Buccal capsule with a ventral pair of prominent semilunar plates or lips, similar to U. Stenocephala, and a dorsal pair of slightly developed lips, of the same nature; dorsal conical median tooth projects prominently into the buccal cavity, similar to Monodontus; one pair or dorsal and one pair of ventral submedian lancets deep in buccal capsule. Male, 7 to 9 m m. long;

*Read before the Georgia Medical Association, Fitzgerald, April 15-16-17, 1908.

that two of these were discovered during an attack of typhoid fever.

The symptoms of the disease usually vary in proportion to the number of offending parasites and to the length of time they have been in the individual, although we occasionally find intense symptoms with mild infection. They usually present themselves to you for treatment for one of four things—indigestion, bronchitis or consumption, weakness or swelling of their ankles.

You find them sallow, tongue and conjunctive pale—muscles flabby and soft though they seem to have lost none in weight.

All have a "Pot belly." There is usually a slight haemic murmur over the heart and coarse rales heard over the lungs. Their faces are pale and haggard and they come in looking like the "last rose of summer." They tell you they are easily tired, have no energy, they eat a large quantity and every fall they give out completely. Usually they suffer from pains in their epigastrium—constipation and diarrhoea alternating. The feces are usually slightly reddish in color although it may have any appearance and contain the ova.

If in children their physical development is below par. In young women they suffer from amenorrhoea or dysmenorrhoea. 356 of the 408 treated gave history of repeated attacks of ground-itch—mazamorra.

It is the exception for one to perspire. The temperature has in all uncomplicated cases been subnormal, pulse always rapid and feeble.

The diagnosis of no disease is so easy and sure. You have but to look at one suffering with this infection to make you suspect it and a simple examination of the feces under the microscope will tell the tale. The presence of the egg in the feces is conclusive.

It is well enough to call attention to the fact that this disease may exist in connection with many other diseases and care should be exercised to not attribute all symptoms to the Hookworm without due consideration.

As to treatment..—The preparation of the patient is essential to satisfactory results. Any means of completely unloading the small intestine is satisfactory—the object being to reach the worm by means of some anthelmentic.

caudal bursa with short dorso-median lobe, which often appears as if it were divided into two lobes and with prominent lateral lobes united ventrally by an indistinct ventral lobe; common base of dorsal and dorso-lateral rays very short; dorsal ray divided to its base; its two branches being prominently divergent and their tips being bepartite; spicules long and slender. Female, 9 to 11 m. m. long; vulva in anterior half of body, but near equator. Eggs, ellipsoid, 64 to 76 micron long by 36 to 40 micron broad, in some cases partially segmented in utero, in other (rare) cases containing a fully developed embryo when oviposited."

Under favorable conditions of heat and moisture, both being necessary, the ovum hatches out in about 24 hours. Each ovum producing one worm.

Fortunately these favorable conditions do not exist in the alimentary canal of a human being, so the number of parasites contained depends entirely on the number that gain entrance from without. In about 5 days the larvae reach maturity and is encysted. This is its infective stage. It remains thus dormant until it gains entrance to its human abode where it is said to reach full size and maturity in about five weeks.

There are two modes of infection—direct ingestion of the larvae peros and indirect by finding their way through the skin to the veins and lymphatics and thence to the intestine. For a more thorough explanation see "Report of Commission for the Study and Treatment of Anemia" in P. R. 1904.

Moist, sandy, warm and shady places are the best fields for the development of the larvae. These conditions exist about many gardens and around "horse-lots," and homes. There, on the vegetables ofttimes lies the dormant larvae and when ingested he readily finds a welcomed home. The children often play, barefooted, around the "horse-lots" and grounditch,—mazamorra—a mere symptom of the infection, is very common. Then there are a few scattered here and there who are dirt eaters and they get a thorough charge of the "Hookworm."

Out of 408 treated by me during the past four years, 302 were males—181 whites, blacks, 121. Of the males all had history of repeated attacks of ground itch—mazamorra. Of the 106 females, 100 were blacks and 6 whites. 54 gave history of ground itch—mazamorra. It might be interesting here to state

I usually keep the patient under treatment for 24 hours—allow them to take a glass of milk for dinner—at about 2 p. m. give them 2 to 4 grains calomel, at 4 p. m. repeat the calomel. Allow them to drink water and a cup of coffee or tea for supper. At 9 p. m. give dose of salts or a seidlitz powder. At 5 a. m. take from 15 to 20 grains of betanaphthol in powder followed by 1-2 or 1 glass of water, at 7 repeat the betanaphthol. At 11 a. m. give a seidlitz powder or dose of salts. At 12 m. give milk and allow them to gradually resume their former diet.

The first 150 cases were treated with thymol, but the danger of the drug and its intolerance by many, led me to try something else. My results from betanaphthol have been absolutely satisfactory.

Instructions are always given that no alcoholic stimulants be used or any oils given.

A second or third treatment is rarely necessary. As the parasite lives only a few years in the intestine and a few do no serious damage—provided only a few remain and the sufferer improves—it is unwise to force them to submit to several treatments. If in two months they have not improved satisfactorily it would be well to investigate and if wise treat them again.

Tonics containing iron and strychnine were given to every other one of the first 100 treated. The progress was no more satisfactory then those who took nothing, so I have discarded after treatment only to advise as to the best articles of diet.

As repeated infections are quite common it is well to warn them of that danger. For this purpose I copy the instructions given by the Porto Rican Commission, which are—"Have a privy in your house. Do not defecate on the surface of the ground, but in the privy. Do not walk barefooted so that you may avoid contracting (grounditch) mazamorra in your feet. Wear shoes and you will never suffer from anemia."

---

Just preceding the meeting of the American Medical Association, the American Medical Editors' Association will hold its annual session at the Auditorium, Chicago, on May 30th and June 1st. An interesting programme has been prepared. Medical editors not members of the Association, are invited to attend.

